# Supplementary material for: Health promotion and disease prevention registries in the EU: a cross country comparison
Source: Arch Public Health. 2023 May 10;81:85. doi: 10.1186/s13690-023-01097-0 (PMC10170815; doi:10.1186/s13690-023-01097-0)
Supplement: Supplementary file 1 — Supplementary Material 1 [file 13690_2023_1097_MOESM1_ESM.pdf]

Health Promotion and Disease Prevention Registries in the E.U.: A cross country comparison

Statement

What is known

Health promotion and disease prevention programme registers (HPPR) can play an important role in increasing transparency in the assessment and selection of effective and efficient practices, and encourage further implementation of such practices to improve health promotion and disease prevention efforts. These registries serve as entry points and practical repositories, enabling decision-makers to have easy access to (evidence-based) practices. There are different types of national or transnational registries, ranging from comprehensive registries (e.g., *"Best or Good Practice Portals"*) to more specific thematic registries (e.g., the portal of the European Monitoring Centre for Drugs and Drug Addiction (EMCDDA)). Earlier studies on programme registers focused on topical, evidence-based programme registers with an emphasis on matters of assessment (including rating paradigms and evidence schemes (1-3). However, understanding the implementation of assessed practices included in national HPPR requires a holistic analysis of the registers and how they are functioning to promote the use of recommended practices. To date, there has been no comparison or overview of either: 1) national HPPR across Europe and 2) national HPPR with reference to the European Context (the EU Public Health Best Practice Portal).

What we add

In this study, we extend the evidence on programme registers by giving insight into core structural elements of programme registers operating on a (trans)national level. This includes a comparative overview of the various approaches taken in several national HPPR (and one transnational HPPR) and identification of commonalities and differences in the mechanisms the included HPPRs use for assessment, classification, designation, and implementation of practices. The results of this study indicate that all HPPRs share the overall aim of selecting, providing, and promoting recommendable health promotion and disease prevention practices, but differ methodologically in the studied criteria. While all HPPRs collect and share recommendable practices, others have implemented further measures to improve the quality of submitted practices. Collaboration between HPPRs (at national and EU levels) is appreciated, especially in terms of using consistent terminology to avoid misinterpretation and facilitate cross-country comparison, as well as to facilitate discussion on the adaption of assessment criteria by national HPPRs. Greater efforts are needed to promote the actual implementation and transfer of effective practices on national level in order to address public health challenges with these practices.

Implications for public health

Several national HPPRs have been developed across Europe and others are currently under development. This is a welcome development as it means that more institutions on the state level have recognized the need and added value of this approach to promote the implementation of

(evidence-based) health promotion and disease prevention practices. This study provides a starting point for research in the area of national HPPR in the European context by comparing various approaches in the functioning of current national HPPR. The results of this study may be informative in guiding the development of national program registries in countries with no or starting HPPR, or for further development of existing HPPRs across Europe. Further, a coordinated and consistent approach across the EU in identifying, collecting and analysing health promotion and disease prevention practices can enable cross-national comparisons, more effective knowledge transfer and faciliate joint efforts to combat public health issues that often do not respect national borders.

1. Burkhardt JT, Schröter DC, Magura S, Means SN, Coryn CL. An overview of evidence-based program registers (EBPRs) for behavioral health. Eval Program Plann. 2015;48:92-9.
2. Means SN, Magura S, Burkhardt JT, Schröter DC, Coryn CLS. Comparing rating paradigms for evidence-based program registers in behavioral health: evidentiary criteria and implications for assessing programs. Eval Program Plann. 2015;48:100-16.
3. Zack MK, Karre JK, Olson J, Perkins DF. Similarities and differences in program registers: A case study. Eval Program Plann. 2019;76:101676.
