# Supplementary material for: Health promotion and disease prevention registries in the EU: a cross country comparison
Source: Arch Public Health. 2023 May 10;81:85. doi: 10.1186/s13690-023-01097-0 (PMC10170815; doi:10.1186/s13690-023-01097-0)
Supplement: Supplementary file 2 — Supplementary Material 2 [file 13690_2023_1097_MOESM2_ESM.docx]

**Manuscript Health Promotion and Disease Prevention Registries in the E.U.: A cross country comparison**

Ref: Submission ID 738a5704-d382-4db6-8b26-c080441ae274

Dear reviewer,

We thank you very much for your valuable comments on our manuscript in which we compared six HPPRs in Europe. We have used most of your suggestions to improve our manuscript.

A major comment was the readability of our manuscript. We have asked a native speaker to correct the manuscript and we hope this have improved the quality of our text.

In the attachment we describe the changes we have made and an explanation why we didn’t applied the suggested change.

We look forward to your decision.

Kind regards,

Dr. D. van Dale

[Djoeke.van.dale@rivm.nl](mailto:Djoeke.van.dale@rivm.nl)

+31-629601801
