# Supplementary material for: Health promotion and disease prevention registries in the EU: a cross country comparison
Source: Arch Public Health. 2023 May 10;81:85. doi: 10.1186/s13690-023-01097-0 (PMC10170815; doi:10.1186/s13690-023-01097-0)
Supplement: Supplementary file 3 — Supplementary Material 3 [file 13690_2023_1097_MOESM3_ESM.docx]

**Health Promotion and Disease Prevention Registries in the EU: A Cross Country Comparison**

Christin Rossmann^1^, Sandra Radoš Krnel^2^, Marika Kylänen^3^, Katarzyna Lewtak^4,5^, Claudio Tortone^6^, Paola Ragazzoni^6^, Mara Grasso^6^, Alison Maassen^7^, Luciana Costa^8,9^ and Djoeke van Dale^10*^

^1^ Federal Centre for Health Education (BZgA), 50825 Cologne, Germany

^2^ National Institute of Public Health, 1000 Ljubljana, Slovenia

^3^ Finnish Institute for Health and Welfare (THL), PO Box 30, 00271 Helsinki, Finland

^4^ National Institute of Public Health NIH-NRI (NIPH NIH-NRI), 00-791, Warsaw Poland

^5^ Medical University of Warsaw, 02-007 Warsaw, Poland

^6^ DoRS – Health Promotion Regional Documentation Centre, Regione Piemonte ASL TO3, I-10095 Grugliasco (Turin), Italy

^7^ EuroHealthNet, 1000 Brussels, Belgium

^8^ National Institute of Health Dr. Ricardo Jorge, 1649-016 Lisboa, Lisbon Portugal

^9^ BioISI–Biosystems and Integrative Sciences Institute, Faculty of Sciences, University of Lisbon, Portugal

^10^ National Institute for Public Health and the Environment, PO Box 1, 3720 Bilthoven, The Netherlands

*Correspondence: djoeke.van.dale@rivm.nl

**Abstract**

**Background**: Health promotion and disease prevention programme registries (HPPRs), also called ‘best practice portals’, serve as entry points and practical repositories that provide decision-makers with easy access to (evidence-based) practices. However, there is limited knowledge of differences or overlaps of current national HPPRs in Europe function, the context and circumstances in which these HPPRs were developed, and the mechanisms utilised by each HPPR for the assessment, classification and quality improvement of the included practices.

This study prepared an overview of different approaches in several national HPPRs and the EU Best Practice Portal (EU BPP) as well as identified commonalities and differences among the core characteristics of the HPPRs.

**Methods:** We conducted a descriptive comparison – that focused on six European countries with existing or recently developed/implemented national HPPR and the EU BPP –to create a comparative overview. We used coding mechanisms to identify commonalities and differences; we performed data management, collection and building consensus during EuroHealthNet Thematic Working Group meetings.

**Results:**  All HPPRs offer a broad range of health promotion and disease-prevention practices and serve to support practitioners, policymakers and researchers in selecting practices. Almost all HPPRs have an assessment process in place or planned, requiring the application of assessment criteria that differ among the HPPRs. While all HPPRs collect and share recommendable practices, others have implemented further measures to improve the quality of the submitted practices. Different dissemination tools and strategies are employed to promote the use of the HPPRs, including social media, newsletters and publications as well as capacity building workshops for practice owners or technical options to connect citizens/patients with local practices.

**Conclusions:**

Collaboration between HPPRs (at national and EU level) is appreciated, especially regarding the use consistent terminology to avoid misinterpretation,facilitate cross-country comparison and enablediscussions on the adaption of assessment criteria by national HPPRs. Greater efforts are needed to promote the actual implementation and transfer of practices at the national level to address public health challenges with proven and effective practices.

**Keywords:** evidence-based practices, health promotion, disease prevention, implementation, assessment

**Background**

The value of building on the best available evidence and adopting cost-effective practices known to achieve better health and wellbeing outcomes for individuals, communities and populations has been widely acknowledged in research, policy and practice (1, 2). The availability and access to those practices may reduce the implementation of inferior or even counterproductive practices and the temptation to “reinvent the wheel”, eliminatingpossible mistakes in developing new interventions (3). To ensure the implementation of these practices, it is essential that decision-makers can easily access information on effective and efficient practices, are familiar with the available evidence and know how to put it into practice (4, 5).

Health promotion and disease prevention programme registries (HPPRs), commonly referred to as “Best or Good Practice Portals”, can play an important role in increasing transparency of effective and efficient practices to support the decision for an adequate practice of a health problem and thus, in implementing the practice. These registries serve as entry points and practical repositories, giving decision-makers easy access to (evidence-based) practices (6). Because many decision-makers prefer information in their own language and approaches that are optimally suited to their national or local contexts, several national arose across Europe and others are currently under development. This is a welcome development as it means that more institutions on the state level have recognised the need and the added value of this approach and are trying to promote the implementation of health promotion and disease-prevention practices.

Although one can justify each national approach and has its merits, the Joint Action On Chronic Diseases (CHRODIS) has advocated for more evidence-based practices by jointly organizing a way of implementing them in the European Union (EU). A coordinated and consistent approach across the EU in identifying, collecting and analysing health promotion and disease-prevention practices can enable cross-national comparisons (7), more effective knowledge transfer and joint efforts to combat public health issues, which often do not respect national borders. To what extent to which these goals might be achieved, however, is unclear. Indeed, there is limited knowledge of the extent to which there are informative differences or overlaps in the functioning of current national HPPRs in Europe, the context and circumstances in which they were developed, and the mechanisms chosen for assessing, classifying them and improving their quality.

Therefore, we aim to establish a starting point for research in this area by providing a comparative overview of the various approaches in several national HPPRs and one transnational HPPR that currently exist in the EU, and to identify commonalities and differences in mechanisms within the HPPRs in terms of the assessment process and criteria, classification, designation and implementation. The results of this study may be informative for guiding the development of similar resources in other countries to strengthen health promotion and disease prevention efforts throughout the EU.


**Methods**

We conducted a descriptive comparison toprovide an overview of HPPRs while using coding mechanisms to identify commonalities and differences.

## **Definition of key terms**

In the literature broadly accepted definitions for general key terms were either lacking or were applied differently.. Three working group members adopted or adapted the definitions for these terms and discussed them in a meeting with the entire group to reach a consensus.

Table 1 Definition of general key terms

| **Term** | **Working definition -/ explanation** | **Citation** |
| --- | --- | --- |
| Health promotion and prevention programme registry (HPPR) | “(1) web-based collections of (2) health promotion and disease prevention interventions that use (3) documentable criteria for including and excluding programmes or interventions, and (4) [that] feature evaluative information that could support decision making.” | Burkhardt et al. (8) |
| “Best” practice | “Best Practices […] have been shown to be effective in improving the health of the population when implemented in a specific real-life setting and are likely to be replicable in other environments” | Ng and de Colombani (3) |
| “Good” practice | Focused on process (how an intervention and its associated effects emerge, adapt and perform in relation to a particular time, space and practice of local implementation) rather than outcomes (assumed isolatable effects of particular interventions into implementation). | Adapted from Barnfield et al. (9) |
| Practice | An umbrella term for interventions, projects and programmes that are included in Health Promotion and Disease Prevention Registries | Working definition established by members of the working group for the purpose of this article |

**Setting**

To access information and first-hand-knowledge about the various HPPRs, we established a working group on best practice portals was in 2019 within EuroHealthNet, a not-for-profit partnership of public institutions operating at local, regional, national and international levels across Europe (10). EuroHealthNet supports members’ work through project development, knowledge exchange, capacity building, policy monitoring, policy development, research, and communication. The working group comprised public health experts representing public health institutes in Germany, Finland, Italy, the Netherlands, Poland, Portugal, and Slovenia (and collaborating with DG SANTE/EU Joint Research Centre representatives for the European HPPR). The members exchanged experiences, presented and compared national HPPRs and learned from each other. Further, they also explored opportunities for greater linkages and synergies between European-level HPPRs and national HPPRs to promote evidence-based practice in public health across countries.

**Data sources**

This study focuses on six European countries Germany [DE], Italy [IT], The Netherlands [NL], Finland [FI], Poland [PL], and Slovenia [SI] had an existing ([DE], [IT], [NL]) or recently implemented ( [FI], [PL], Slovenia [SI]) national HPPR and included the European Public Health Best Practice Portal [EU] benefitting the entire EU. The parent organisations responsible for creating and managing each portal (Table 2) are often national health promotion and public health research and development. The Public Health Best Practice Portal developed by the European Commission (DG SANTE) and the Italian “Pro.Sa database”, developed by a regional organisation but implemented nationally, are exceptions to this finding.

All included HPPRs share the common strategy of creating an easily accessible, centralized database platform of health promotion and disease-prevention practices to select, provide and/or share practices for professionals, policy makers, decision-makers on different levels,as well as students, researchers and non-governmental organisations (NGOs)in the field of health promotion and disease prevention. All HPPRs offer a broad range of health promotion and disease-prevention practices, including practices that should be implemented in specific settings (e.g., schools, community), for specific target groups (e.g., children, older adults, socially disadvantaged groups) and in various thematic areas (e.g., mental health, nutrition, health policy). Some portals (EU, Finland and Slovenia) have a broader focus and include additional topics such as end of life palliative care (EU), culture and education (Finland) and public health (Slovenia).

Table 2: Organisational characteristics of Health Promotion and Prevention Registries

| **Country/**  **Continent** | **Europe (EU)** | **Finland (FI)** | **Germany (DE)** | **Italy (IT)** | **Netherlands (NL)** | **Poland (PL)** | **Slovenia (SI)** |
| --- | --- | --- | --- | --- | --- | --- | --- |
| **Responsibility**  The parent organisation responsible for building and maintaining the registry | Steering Committee for Health Promotion and Disease prevention | Finnish Institute for Health and Welfare (Terveyden ja hyvinvoinnin laitos, THL) | Federal Centre for Health Education (Bundeszentrale für gesundheitliche Aufklärung, BZgA) and Network of Equity in Health (Kooperationsverbund Gesundheitliche Chancengleichheit) | Health Promotion Documentation Centre, Piedmont Region (Centro Regionale di Documentazione per la Promozione della Salute, DoRS) | National Institute for Public Health and the Environment (Rijksinstituut voor Volkgsezondheid en Milieu, RIVM) | National Institute of Public Health NIH – National Research Institute (Narodowy Instytut Zdrowia Publicznego PZH - PIB) | National Institute of Public Health (Nacionalni inštitut za javno zdravje, NIJZ) |
| **Name** | Public Health Best Practice Portal | Hyvinvointia ja terveyttä edistävien toimintamallien arviointi | Praxisdatenbank | Pro.Sa database | Loketgezondleven.nl | ProfiBaza | Portal za izmenjavo primerov dobrih praks na področju javnega zdravja |
| **Website** | <https://webgate.ec.europa.eu/dyna/bp-portal/> | <https://thl.fi/hytearviointi> | <https://www.gesundheitliche-chancengleichheit.de/praxisdatenbank/> | <https://www.retepromozionesalute.it> | <https://www.loketgezondleven.nl/interventies-zoeken#/overview> | https://profibaza.pzh.gov.pl/ | <https://www.nijz.si/publikacije/merila-za-vrednotenje-intervencij-na-podrocju-javnega-zdravja> |
| **Start date**  Year in which the HPPR was launched | 2016 | 2011 | 2003 | 2001 | 1999 | 2021 | 2020* |
| **Funding**  Funding source (organisation) | EU Commission; Directorate General for Health and Food Safety (DG Santé) | Finnish Institute for Health and Welfare (THL). Funding by the Sustainable Growth Programme for Finland (RRP) for the digital development of the new HPPR (2022-2024). | Federal Centre for Health Education (BZgA) | Department of Health, Regional Government of Piedmont and additional episodic funding by Italian Ministry of Health | Ministry of Health, Welfare and Sports | Co-financed by the European Union from the European Regional Development Fund for 2014-2020, from 2021 funding by Ministry of Health | Ministry of Health Slovenia |

*Year in which the criteria for evaluating public health interventions were published and active development of the HPPR started

**General characteristics of the EU Public Health Best Practice Portal and Health Promotion Prevention RegistriesEurope:** The Public Health Best Practice Portal was launched in 2016 following the priority established by DG SANTE to identify, disseminate and transfer “Best Practices” to improve the implementation of best practices in Europe. In this way, DG SANTE hopes to achieve Sustainable Development Goal 3.4 to reduce premature mortality from non-communicable diseases by one-third by 2030 through prevention and treatment, and to support the achievement of the nine UN/WHO voluntary global health targets. A Steering Group on Health Promotion, Disease Prevention and Management of Non-Communicable Diseases is the mainstay of its organisational structure.

**Finland:** The Finnish Institute for Health and Welfare started the first national HPPR called Innokylä in 2011 to collect and facilitate the development of (evidence-based) practices. A new Finnish HPPR, Hyvinvointia ja terveyttä edistävien toimintamallien arviointi, started the assessment of evidence-based practices in 2019. The first steps included developing and piloting of the assessment process and criteria. In 2022-2024, THL will develop a new digital HPPR. The objectives of the evidence-based programme registry are to (1) assess and publish evidence-based practices in health and well-being promotion; (2) facilitate comparison of effectiveness, evidence, and transferability of practices; (3) improve knowledge management in health and well-being promotion.

**Germany** The HPPR of Germany is one element of a network (Gesundheitliche Chancengleichheit), initiated in 2003 by the Federal Centre for Health Education (BZgA) to address social and health inequity. They introduced the assessment criteria (“good practice criteria”) primarily as a self-reflection tool for professionals in municipalities that want to further develop their practice(s) in terms of quality and sensitivity towards social health inequality. The objectives of the Germany’s HPPR are to: (1) provide an overview of community practices that address social and health inequities; (2) support knowledge exchange and communication, and to create transparency among practice actors; (3) showcase exemplary practices.

**Italy** The HPPR of Italy, Pro.Sa - Prevention and Health Promotion Projects and Interventions Database (Pro.Sa - Banca dati di Progetti e Interventi di Prevenzione e Promozione della Salute), was officially launched in 2001 by DoRS, the Health Promotion Documentation Centre of the Piedmont Region. The objectives of Pro.Sa are to (1) collect, monitor and share projects, interventions, programmes and “transferable good practices”, (2) evaluate, highlight and disseminate “transferable good practices” in other contexts, (3) support social practitioners and health professionals, decision-makers and stakeholders in decision-making regarding strategies, planning, and evaluation of health promotion and prevention. In this way, Pro.Sa provides an overview of practices by documenting all regional and local projects and experiences developed under the Regional Prevention and Health Promotion Plan and other programmes, highlighting ’transferable good practices’that could be disseminated and implemented in other contexts.

**The Netherlands:** In 1999, the National Institute of Health Promotion and Disease Prevention for the collection of practices. To gain insight into the quality of health promotion and disease-prevention practices,in 2007 the National Institute for Health and Environment ( in collaboration with other health institutes on behalf of the Ministry of Health, Sports and Welfare ) introduced an assessment system to evaluate the quality, effectiveness and feasibility of practices. It applied the system in six domains: health promotion, youth (health) care, sport and exercise, social domain, long term care of older people and mental health care. The objectives of the Dutch HPPR are to (1) improve the quality of health-promotion interventions in The Netherlands, (2) provide insight into the quality, effectiveness and feasibility of health promotion-interventions, (3) create an’upward pressure’ in quality development of health promotion (from practice- to evidence-based interventions).

**Poland:** The idea behind the ProfiBaza Portal – launched in late 2021 - was born in 2016, when information about implemented health programmes was still scattered and unavailable in Poland via open access. This included collecting information about ongoing practices, summarising them in one place and making the data available to a wide audience.. The objectives of the Polish HPPR include(1) assisting the planning, implementation and evaluation of public- healthinterventions in Poland as well as promoting multisectoral collaboration in health and addressing social inequalities in health, (2) building a platform for knowledge translation as part of the “ProfiBaza” system, (3) improving the quality of public-health interventions implemented in different settings as well as the quality of collected data on health-promotion interventions carried out in Poland.

**Slovenia:** The starting date for the Slovenian HPPR was in 2020 with the publication of the report “Criteria for evaluating Public-Health interventions for the purpose of identifying and selecting good practices”, a questionnaire for collecting good practices and the methodological guidelines for evaluating practices together with the evaluation form. The National Institute of Public Health (the lead institution) and the Faculty of Social Sciences of the University of Ljubljana are involved in developing o the HPPR. Unlike other existing websites in Slovenia that focus only on collecting various public heath interventions, the Slovenian portal aims to establish a system for recognizing examples of good practices and to promoting the use of these approaches in the public health. The objectives of the Slovenian HPPR are to: (1) raise the standards of public-health interventions and to improve their quality, (2) provide an overview on quality and effectiveness of public health interventions, (3) support knowledge exchange and the use of effective approaches by providing a pool of reviewed interventions.

**Data management and collection**

We performed data collection (i.e., information on organisational issues, users, resources, assessment process and criteria, presentation of interventions and usability, dissemination and implementation of good and best practices,) in several steps during regular meetings with the working group over a period of 18 months.. To make comparisons, we first identified the characteristics of HPPRs through a review of the literature regarding other programme registries (6, 8, 11-13), evidence-based practice (2, 14) and implementation of practices by programme registries (15). We then listed and discussed the identified characteristics in the group, with each characteristic reviewed to determine its usefulness in comparing the core elements of HPPRs. We considered each selected characteristic to represent an important processual element used in HPPRs or one that might influence the implementation of health-promotion or prevention-related practices. Consensus was reached when more than 80% of participants strongly supported either the inclusion or exclusion of a characteristic. The working group chose and agreed on four characteristics (Table 3)These were: “assessment process”, “assessment criteria”, “incentives for submission and implementation of practices” and “dissemination”. For a more detailed analysis and a clearer overview, the assessment process was divided into: “method of the assessment process”, “result and classification of assessment process” and “designation of practices”.

**Data elements**

Three members of the working group developed working definitions for each characteristic, which were discussed in plenum meetingsuntil consensus was reached. Where possible, we used the work of other authors to develop a set of working definitions. The entire working group either adopted the definitions or adapted them to ensure clarity (Table 3). Once consensus had been reached, the data on each characteristic provided by the working group members representing each country (or the EU) for each HPPR were organised in a spreadsheet format.

Table 3. Working definition of characteristics

| **Characteristic** | | **Working definition** |
| --- | --- | --- |
| **1.** | Assessment process |  |
| 1.1. | Method of the assessment process | A stepwise process that examines practices according to specific assessment criteria. A result is often the inclusion or exclusion of the practice in the programme registry or the classification of the practice. The assessment process is either executed before or after the practice has been integrated into the registry. The general aim behind the assessment is to ensure the implementation of practices, that work. |
| 1.2 | Assessment criteria | Registry-related indicators, that are relevant to an assessment and for the distinction of practices in HPPR |
| 1.3 | Result and classification of assessment process | The grouping of a practice after the assessment process, which is linked to the fulfilment of certain assessment criteria in an HPPR |
| 1.4 | Designation of practices | The final naming/titling of an assessed practice |
| **2.** | Incentives for submission or implementation of practices | Motivational strategies to encourage users of the HPPR to submit their practice to the HPPR or to implement existing practices from the HPPR |
| **3.** | Dissemination | “Dissemination is an active approach of spreading [practices] to the target audience via determined channels using planned strategies“* |

*Adapted definition from Rabin, Brownson (16), consented by working group


**Analysis**

We used a descriptive comparative approach to provide an overview of the various procedures and processes used by the HPPRs studied.

Specifically, to identify common or divergent themes, we analysed the data using coding mechanisms based on our previously developed concept of characteristics (see data management and collection). Two working group member independently summarized the key messages for each characteristic (in narrative form) to confirm that these messages were accurate and understood consistently. These summaries were then presented to the entire group and disagreements in interpretation or wording were discussed (and clarifications made) until consensus (agreement for >80% of the group) was reached.

**Results**

We present the key results in three subsections: 1 Assessment process, 2 Incentives for submission and implementation and 3. Dissemination (Table 3).

The assessment process is a stepwise procedure that examines practices according to the specific assessment criteria. Thus, the core elements of the assessment process are the HPPR criteria (registry-related indicators, relevant to an assessment and distinction of practices), the resulting classification of practices and the way practices appear in the registers (designation of practices). The implementation and dissemination of the included best practices are related to the assessment process.

#### **Assessment process**

Almost all HPPRs have a practice assessment process in place or planned, which required the application of assessment criteria. In all programme registries, practice recording is done through submission of the practice by the practice owner, except in Germany, where a practice must instead be recommended by the coordination office of Equity in Health before it is reviewed and described in detail. All HPPRs, except Poland, where this process has not yet been implemented, use external reviewers (two to three individuals) to execute the assessment. For the final assessment, some HPPRs (NL, SI, EU) use consensus meetings with an external committee of reviewers representing science, policy and practice.

##### **1.1 Method of the assessment process**

Most of the HPPRs included (EU, FI, IT, SI) use a scoring system, whereby a practice must achieve a pre-determined score in successive assessment levels before receiving a classification or being classified as a recommendable practice, Figure 1 shows an example of the EU HPPR point-grading assessment system.). The selection process for the EU HPPR consists of three steps. The first step is the inclusion or exclusion of the submitted best practice including consideration of the political and strategic relevance of the practice, evaluation of ethical aspects and the possible conflict of interest, the description of the intervention (such as identification of the target population, objectives and methodology), and evidence and theoretical underpinning to ascertain the evidence- and theory-based approach. Inclusion in the portal practices requires a minimal score. If the practice is included, the next step is the evaluation of the effectiveness and efficiency of the practice, as well as how the practice addressed equity issues. Finally, the last step focusses on the information about its transferability to other settings and contexts (e.g., availability of manuals, intervention material, training and necessary actions to overcome barriers), sustainability, ability to foster collaboration among different sectors and the inclusion of stakeholders through the whole cycle of the practice (17).

In Finland, Germany, The Netherlands, Italy and Slovenia, there is close personal contact between the practice owner and advisors or evaluators during the assessment process. In these countries, the process is seen as a learning process in developing and implementing practices. For example, in Germany, practice owners can use the assessment criteria (“good practice criteria”) as a self-reflection tool to develop and improve the quality of their practice (e.g., focus on reducing social health inequalities). If the advisors of the the Equity in Health Coordination Office recommend the practice, internal experts conduct an in -depth interviews with the practice owner to qualitatively whether and to what extent the criteria have been met. In The Netherlands and Finland, an assessment form guides the reviewer or the advisor, who comment and suggest improving the practice. During the process, the practice owners and advisors stay in regular contact via email to share experiences and give advice on improving the practice. The advisors support the practice owners in submitting their intervention. After the final assessment by the external reviewers, the practice owner receives a final general comment on the strengths and weaknesses of the practices and suggestions for improvement. The Italian HPPR has a specific section called “Project Guide” that explains the HPPR assessment criteria and provides examples and suggestions to offer practice owners a practical support in self-reflection and to facilitate the exchange between practice owners and evaluators.
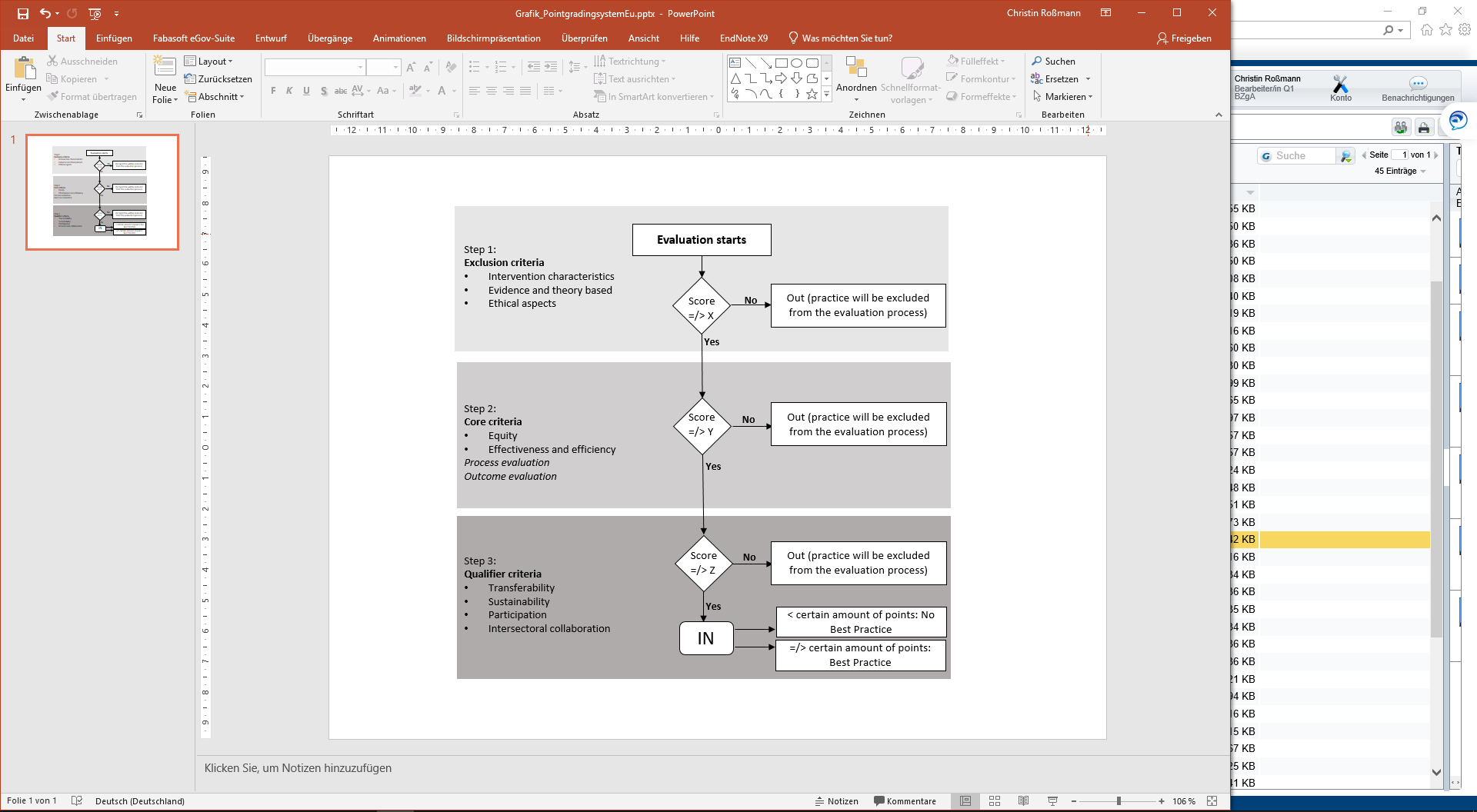


Figure 1 Example of the scoring system by the European Best Practice Portal (adapted ).

#### **1.2 The Assessment criteria**

The assessment criteria of the portals have many similarities.. In many countries, the evaluation criteria consist of three to four main sections and several sub-sections containing more specific criteria. Most HPPR evaluation criteria include: the description of the practice (background, aim, target group, approach/method), theoretical evidence, evidence of the effectiveness of the practice, and its transferability and applicability.

Because of practical implementation of a practice, DE, FI, IT and NL set criteria for the HPPRs, which relate to project design, context or feasibility. Additionally, it is important to note that FI and DE sets criteria focussed on improving the quality and sensitivity of practice to (social) health inequalities.

In some of the HPPRs (EU, FI, SI), assessment of effectiveness is a prerequisite to admitting a practice to the assessment process. In the NL and IT, evaluation of effectiveness is not a prerequisite for the admission to the assessment process, but is needed when it comes to classifying a practice as an “effective practice” (NL) or “transferable good practice” (IT). In the DE, evaluation is not a prerequisite for inclusion in the HPPR or designation as a “good practice”. Interestingly, the EU and Finland are the only registries that require a practice to be demonstrated as cost-effective to be considered as “best practice”.

###### **1.3 Result and classification of the assessment**

The results of the assessment processes vary among the included HPPRs. The process leads to the final inclusion (or exclusion) of the practice in the register in all cases except Germany. If the practice is included, one outcome of the assessment process may be the direct designation of the practice as “best practice” [EU], “transferable good practice” [IT], gGood practice” [SI] or a classification into different levels of evidence (FI, NL) (see Figure 2 for the Dutch example). In Germany, practices are only comprehensively assessed and labelled as good practice” if considered a flagship practice by the Equity in Health coordination office. There is no exclusion of practices from the German register if they fail to meet the assessment criteria. Poland, does not yet have an assessment process .

Finland and The Netherlands practice the classification of practices into different categories.. In the Dutch HPPR, practices meeting the threshold for inclusion in the register are classified into one of three groups (well described, theoretically sound, effective) (Figure 2). In the Finnish HPPR, included practices fall into one of five levels of evidence. In FI, currently the assigned classification is considered ‘final’ (though practice owners can resubmit practices after a few years), whereas the Dutch classification system represents a mechanism to support the practice owner in further developing and improving the practice. During the process, practice owners regularly receive professional advice and recommendationsfrom the Recognition Committee, which may lead to a reclassification of the practice (Figure 2).


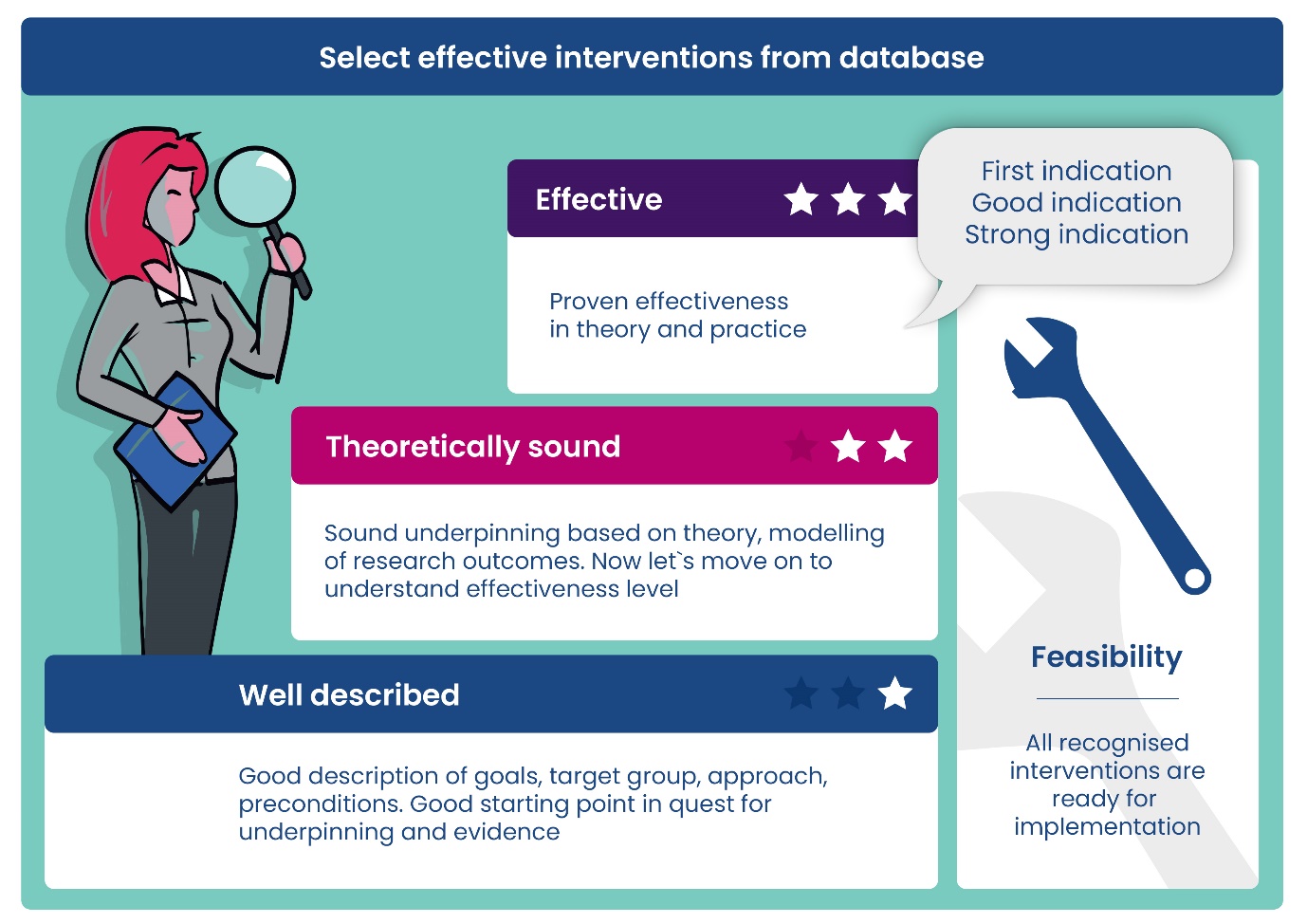


Figure 2: Example of the evidence levels in the Dutch HPPR

##### **1.4 Designation of practices**

Currently, most HPPRs publish their practices with a designation based on the results of the assessment process and the HPPR classification types (see above). Although some HPPRs use the same designation for the “most recommendable” practice (DE, PL, SI, EU, FI), the meaning of this designation is different differs in the variousregistries.

In the German HPPR,for example a “”good ractice“ must meet three assessment criteria ("Good Practice-Criteria"), to be considered from reviewers. The Slovenian and Polish HPPRs use the term “good practice” as well, but they apply (SI) or plan (PL) this designation based on the criteria of the EU Commission, which in turn uses the term bBest practice”. The Italian HPPR uses the term “transferable good practice” for the “best” interventions, meeting a certain score of the register-related criteria that can be transferred and implemented in other contexts. Unlike the other registers, the register in The Netherlands has three designations that describe the classification: well-described (first level), theoretically sound (second level) and effective (third level). Similarly, the Finnish HPPR provides five levels of classification or categorisation, ranging from “poor” to “satisfactory”, “good”, “very good” and “excellent” (Table 4).

#### **2 Incentives for the submission or implementation of practices**

##### *Types of incentives*

##### Incentives to encourage the submission of practices can be explicit or implicit, and include visibility (all HPPRs), feedback and quality improvement (FI, DE, NL, IT) aas well as the possibility to expand the practice (FI, NL and EU).

Through these By the designating/titling practices, practice owners can achieve higher visibility for their practice and may more easily access further funding for their practice. In DE, FI and NL, practice owners receive professional feedback to improve the practice regarding reducing social health inequalities (DE) or its evidence level (NL, FI). In Germany, practices designated as “good practices” are presented as flagship practices, making them much more visible among the other practices included in the HPPR. In Italy, official documents recommending their implementation highlight the “transferable good practices”.

##### *Processes in place for providing incentives*

##### The EU and the Netherlands promote the actual implementation/upscaling of practices. The Netherlands, promotes the submission and implementation of practices from the HPPR in different ways, for example,

- Schools can apply for financial support to implement a recognized intervention.
- The Netherland Organisation of Health Research and Development requires submission of research practices for the Dutch HPPR if they have received a grant to effectively evaluates their practice.
- To implement practices at the regional or municipal level, some municipalities require using practices from the Dutch register.
- The Ministry of Health, Welfare and Sports regularly supports submission with incentive programmes focussing on specific topics missing from the database or implementation programme to support municipalities in implementing “best practices”.

In the EU, practices considered as “best practices” are published on the European HPPR. Best rated practices can be invited to participate in so-called “marketplace events” including the presentation and discussion of the practice with national experts. Once a “best practice” is considered for transfer, it can be selected for implementation of the practice in further Member States. This is done through “Joint Actions” under the EU4Health Programme, which are co-funded by the EU.

#### **Dissemination**

All HPPRs use a comprehensive range of dissemination tools and strategies to promote the use of the HPPRs, such as social media, newsletters and publication of articles.

Finland, Germany, the Netherlands and Poland employ further dissemmination. In Finland and Germany, for example, also promote dissemination through workshops and training programmes conducted to improve the quality of health promotion and prevention practices. Germany offer a guide operationalising the “good practice criteria”. Thehe Netherlands, provides a thematic overview of effective practices is provided in policy briefs, national prevention programmes and manuals (e. g.in the “Healthy School Manual” and “Healthy Municipal Manual”). In Poland, individuals can find opportunities to participate in health-promoting practices in their area by voluntarily integrating practices into the ”Patient Internet Account“(Ministry of Health). In Finland, once the intervention has been accepted for publication, the assessment is published in the portal and archived in the Finnish database “HYTE-toimintamalli”.

Table 4: Assessment process, method, classification and designation

|  | 1. **Assessment process** | **1.1 Assessment method** | **1.2 Assessment Criteria** | | **1.3 Classification and 1.4 Designation of most recommendable practice** |
| --- | --- | --- | --- | --- | --- |
| **Europe** | 1. Call for best practices 2. Submission of practices by countries 3. External evaluation using criteria Steering group | Point-grading-system | - Exclusion criteria (relevance, intervention characteristics, evidence and theory based, ethical aspects), - Core criteria (effectiveness and efficiency of the intervention, equity), - Qualifier (transferability, sustainability, participation, intersectoral collaboration) | | Best practice |
| **Finland** | Open peer review:   1. Submission of the practice by the practice owner 2. The editorial team assesses applicability to the evaluation process 3. The author decribes the practice by filling the evaluation criteria 4. At least two peer-reviewers evaluate the practice and may ask for revision and additional information   If the peer reviews differs widely, the editorial team may invite an additional peer reviewer. | Point-grading-system, including qualitative and quantitative criteria | Evaluation criteria:   - Basic information of the practice and applicability to the evaluation process (for the acceptance to the evaluation process) - Description of the impact chain of the practice (e.g., background, aim, target group, experts and stakeholders, approach/methods, process evaluation and quality assurance, results and effectiveness, costs and cost effectiveness, risks, ethical considerations, management) | - Evidence of the effectiveness and cost-effectiveness of practice (e.g. studies, reports, calculations) - Transferability and applicability of practice (e.g., availability of training material and training, regional transferability, implementation) - In addition, a list of outputs and publications related to the practice | Evidence-based practices:  5- level classification:  1 = poor practice  2 = satisfactory practice  3 = good practice  4 = very good practice  5 = excellent |
| **Germany** | 1. Recommendation (e.g., by the Equity in Health coordination office) 2. Interview with practice owner and description by the staff of BZgA 3. 3 Assessment of practice by two experts of advisory working group of cooperation office 4. 4. After several feedback loops and positive assessment the practice is published in the practice database | Self-reflection,  in-depth-interview with practice-owner | Good Practice Criteria recommended for use:   - Concept and project planning, - Target group orientation - Settings approach - Integrating intermediaries - Sustainability - Low-threshold methodology, | - Participation - Empowerment - Integrated action/networking - Quality management - Documentation and evaluation - Capturing cost effectiveness | Good Practice |
| **Italy** | 1. Submission by the practice owner 2. Two independent reviewers (one expert in health promotion methodology, one expert in topic or setting) evaluates and may ask for revision and additional information to the documents (two or more revisional loops). 3. After the required addictions/changes the practice is published and described in its strengths and limitations and with suggestions for the transferability. | Point-grading-system | Principle and values:   - Equity - Empowerment - Participation   Planning and evaluation:   - Context analysis - Setting - Theories and models - Evidence and good practices | Objectives:   - Description of actions/interventions - Resources, timelines, and constraints - Process evaluation   Impact and outcome evaluation   - Sustainability and transferability: - Partnerships and alliances - Sustainability - Transferability - Communication | Transferable Good Practice |
| **The Netherlands** | 1. Submission by the practice owner 2. Advice on draft description by external advisors to improve quality of submission (2-3 loops) 3. Assessment of the programme by three members of the committee (representatives of practice and science) during a meeting 4. Intervention and the recognition level are presented in the portal with evidence level | Qualitative assessment (criteria provide guidance for evaluator to make suggestions for improvement) | Description of the intervention:   - Objectives - Target group - Involvement of target group - Method/approach - design and content   Theoretical underpinning:   - Problem analysis - Factors addressing the problem - Justification of the method (change theory and empirical evidence) and summary of core elements | Feasibility:   - Training and competencies professionals - Material for implementation - Recruitment and evaluation of the intervention - Quality assurance - Prerequisites for implementation - Resources   Evaluation:   - Process evaluation and effect evaluation: number of studies, methods and results | Recognized interventions:  five-level-classification:   1. Well-described 2. Theoretically sound 3. First indications for effectiveness 4. Good indications for effectiveness 5. Strong indications for effectiveness |
| **Poland** | Planned: The review is done by 3 to 5 evaluators (working group and support from advisory group) | Not introduced so far | Planned: development of criteria to assess submitted interventions based on EU-Commission criteria and those applied in other national programme registries | | Good Practice |
| **Slovenia** | 1. Submission to the database by the practice owner (e.g., questionnaire and all relevant documentation) 2. Assessment of the intervention by three reviewers (representatives of practice and science) 3. First panel meeting of reviewers to prepare additional questions and requests for the intervention owners 4. Peer-reviewers meet with the owners of the intervention, requesting additional information and revisions, as needed 5. 3-4 panel meetings of reviewers to agree upon the final decision regarding the assessment score and to prepare the final document with proposals for possible improvements | Point-grading-system | Assessment criteria adapted from the EU Best Practice Portal and piloted in 2022.  Exclusion criteria   - Relevance of the intervention regarding the objectives of public health policies and strategies - Intervention characteristics and structure - Evidence and theory based - Ethical aspects | Core criteria   - Effectiveness and Efficiency of the intervention - Equity (considering the dimension of equality and equity and efforts to reduce health inequalities) - Participation of target groups and stakeholders - Intersectoral collaboration     Additional criteria   - Transferability - Sustainability | Good Practice |

**Discussion**

For the first time, this cross-county comparison brings together information about six HPPRs in Europe and the EU Best Practice Portal, enabling valuable insights. Our results provide an understanding of the different approaches of the national HPPRs and the functioning of several elements of HPPRs in Europe. They are united in their aim to select, provide and share good or best practices in health promotion and disease prevention to support practitioners, researchers and policy makers in evidence-based decision-making. We reveal differences the existing differences in the classifications, designation of the recommended practices and the dissemination and implementation strategies of the HPPRs.

All HPPRs share the aim to collect, assess and promote “Best” or “Good” practices for health promotion and disease prevention to support practitioners, researchers and policy makers in evidence-based decision making. The majority of the HPPRs are managed by National Public Health Institutes and funded by their Ministries of Health, which reflects the importance and the sustainability of the registers.Some HPPRs (DE,NL,IT) have already existed for more than 10 years and play an important role in evidence-based decision-making in their countries.

Most HPPRs have developed assessment criteria divided into three to four main assessment sections as well as into multiple sub-sections that elaborate the criteria in greater detail to facilitate the assessment,Some HPPRs offer measures that enable practice owners to develop and improve their practice, whether regarding their sensitivity to health inequities, the level of evidence or transferability. This demenonstrates the thegreat priority afforded to implementation of practices (e.g., DE, FIN, IT, NL, SI).

. Promoting evidence-based practices and conducting quality control in health promotion and disease prevention are crucial ensuring the implementation only of effective and efficient practices(18). While this system has its merits, the design of evidence levels and their importance are widely debated among researchers. Means et al. (2015), in their article on rating paradigms for programme registries in behavioural health, noted that registries tend to use a standard hierarchy of evidence (similar to NL and FI), even though health promotion and disease-prevention practices are often complex in nature and require appropriate research designs that may not conform to the standard hierarchy of evidence. Engaging with such complexity requires structures and processes that allow for more flexible research designs and support for different methodologies (19).

Countries differ significantly in how they classify and designate the most recommended practices (e. g., “good practice” [DE, IT, SL], “excellent practice” [FI] or “best practice” [EU]). Should further integration and information exchange be sought between the different European HPPRs, using different definitions, classification methods and designation of practices across the countries may lead to confusion among end-users and misinterpretations of the relative effectiveness of a “good practice” and a “best practice”. Many studies have already noted the variation in standards in programme registries, especially in the course of evidence-based practices (15). In particular, for countries considering the introduction of a national HPPR (e. g., Portugal) or where HPPRs are still under development (SL, PL, FI), adapting the EU assessment criteria (or selecting certain common core criteria) may be a useful solution to promote comparability of practices across countries. For longer existing HPPRs, which already contain specific and unique criteria, adaption may not presently be possible at present, but should be kept in mind in future discussions on the merits of linking national HPPRs and DG SANTE’s HPPR (17).

Additional important findings were in the area of dissemination strategies to promote the use of the HPPRs. Activitiesat the community-level, such as capacity-building workshops for practice owners were recognized as a valuable tool for the dissemination of “good”of “best’ practices.. To reach key decision makers and policy-makers (8), The Netherlands has included an overview of recommended practices by topic in their policy briefs (Manual Health Municipality/- Healthy School and national prevention programmes, to increase the chance of these practices being implemented (18). This approach is in line with the recommendations by Brownson et al. (14), which aim to make evidence more accessible to policy audiences by demonstrating the relevance of public health practices to current policy debates. Simply increasing the visibility of recommendable practices and facilitating their uptake, transfer and implementation, as well as providing financial incentives for implementation are interesting options. In The Netherlands and the EU, the process of selecting and transferring “best practices” is structurally anchored and supported by funding. Both the European and the Dutch HPPR go beyond Brownson et al.’s recommendation (14) by not only sharing information on evidence-based practices but also promoting their implementation through financial support (19). The start of three relatively new HPPRs’(FI,PL,FI) and the development of the European HPPR show the increasing interest in supporting practioners and policymakers with evidence-based information for health promotion and disease prevention with registers or best practice portals. This increasing interest occurs because registers now seen as an effective means of supporting decision-making with evidence-based information (6). Another reason for this increasing interest lies in the rising costs of health care and the need for a shift of focus from health care to health promotion. The European Funding and Innovations Programma Horizon 2021-2027 for example has never had such a large budget for health promotion, disease prevention and tackling the health inequalities (20). The HPPRs are important means for the implementation and sustainability of the results and interventions developed with the framework of Horizon Europe at the European and national level.

**Study limitations and future studies**

This paper reflects on the variety of approaches and elements of select HPPRs across Europe. It could have been beneficial to include all HPPRs in Europe to get a more comprehensive overview of the different approaches and procedural elements. However, including a large number of HPPRs is probably not as important as capturing the diversity of current approaches. Analysing thedifferences how HPPRs are developed and designed may provide valuable lessons or insights, which can support future developments (both in countries with existing portals and those where portals are being developed or considered).

In addition, the selection of characteristics included in this article was relatively narrow designed. HPPRs may be studied from many different angles (e.g., processes for gathering practices, types of practices eligible for inclusion), and with varying degree of intensity (e.g., further in-depth research on dissemination strategies or incentive mechanisms). However, this study intended to provide a starting point for research, which should be followed by further studies on other characteristics that may be important for a successful implementation of HPPRs

**Conclusions**

This study provides valuable information about the the diversity of approaches and elements of selected national HPPRs in Europe. The results of this study indicate that all HPPRs share the overall aim of selecting, providing and sharing recommendable health promotion and disease-prevention practices. While most HPPRs have developed assessment criteria that are divided into three or four main assessment sections, they differ in the methodology the apply to the assessment process, classification and designation of practices. Some HPPRs choose to r focus on collecting and sharing recommendable practices, whereas others have also implemented measures to improve the quality of a practice. Further collaboration between national HPPRs and the EU Best Practice Portal is paramount, especially regarding establishing consistent terminology to avoid misinterpretation, to facilitate cross country comparison, and facilitate discussions on the adaption about the adaptation of assessment criteria by national HPPRs. Greater efforts are needed to promote implementing and transferring recommendable practices at the national level to address public health challenges with recommendable practices. Further research could usefully explore additional aspects of HPPR development and management, and even identify new approaches that support the implementation of practices through HPPRs.

**List of abbreviations**

HPPR: Health promotion and disease prevention programme registries; EU: European Union; NGO: Non-governmental Organisations; DG: Santé Directorate General for Health and Food Safety; THL: Terveyden ja hyvinvoinnin latitos (Finnish Institute for Health and Welfare, Finland); BZgA: Bundeszentrale für gesundheitliche Aufklärung (Federal Centre for Health Education, Germany); DoRS: Centro Regionale di Documentazione per la Promozione della Salute (Health Promotion Regional Documentation Centre); RIVM: Rijksinstituut voor Volksgezondheid en Milieu (National Institute for Public Health and the Environment, Netherlands); NIPH NIH-NRI: Narodowy Instytut Zdrowia Publicznego PZH-PIB (National Institute of Public Health, Poland NIH-National Research Institute); NIJZ: Nacionalni inštitut za javno zdravje (National Institute of Public Health, Slovenia); Pro. Sa: Banca dati di Progetti e Interventi di Prevenzione e Promozione della Salute (Prevention and Health Promotion Projects and Interventions Database, Piedmont Region).

**Declarations**

**Ethics approval and consent to participate:** Not applicable

**Consent for publication:** Not applicable

**Availability of data and materials:** All data generated or analysed during this study are included in this published article

**Competing interests:** The authors declare that they have no competing interests

**Funding:** No funding was requested for this work.

**Authors' contributions**: CR wrote the original draft; CR, SRK, MK, DD and LC with the contribution of all the authors prepared the methodology and analyses; KL. DD, SRK, MK, AM, MG andCTcompleted the visualization as well as reviewed and advised on the original draft. All authors approved the final manuscript and were responsible for the decision to submit for publication.

**Acknowledgements:** The authors would like to acknowledge the exceptional conceptual support and assistance in scientific writing provided by Prof. Dr. Litaker from Case Western Reserve University, Ohio.

**Authors' details**

Christin Rossmann: Federal Centre for Health Education (Bundeszentrale für gesundheitliche Aufklärung, BZgA) Department of Task Planning, Strategy, Knowledge Transfer, International Relations. Cologne, 50825, North Rhine-Westfalia, Germany

Sandra Radoš Krnel: National Expert at National Institute of Public Health, Centre for Analysis and Development of Health. Ljubljana, SI-1000, Slovenia

Marika Kylänen: Finnish Institute for Health and Welfare (THL). Health and Wellbeing Promotion. Helsinki PL 30, 00271 Finland

Katarzyna Lewtak: MD, PhD. 1. National Institute of Public Health NIH _ National Research Institute. Department of Health Promotion and Chronic Diseases Prevention. Warsaw, 00-791, Poland; 2. Medical University of Warsaw. Department of Social Medicine and Public Health. Warsaw, 02-007, Poland

Claudio Tortone: DoRS _Health Promotion Regional Documentation Centre, Regione Piemonte ASL TO3, Grugliasco (Turin) I-10095, Italy

Mara Grasso: DoRS _ Health Promotion Regional Documentation Centre, Regione Piemonte ASL TO3, Grugliasco (Turin) I-10095, Italy

Alison Maasen: EuroHealthNet, 1000 Brussels, Belgium

Luciana Costa: 1.National Institute of Health Dr Ricardo Jorge Department Health Promotion and Prevention Non-Communicable Diseases. Lisboa, 1649-016, Portugal; 2. BioISI – Instituto de Biosistemas e Ciências Integrativas. Faculdade de Ciências, Universidade de Lisboa. Lisboa, 1749-016, Portugal_

Djoeke van Dale: National Institute for Public Health and the Environment, P.O Box 1, 3720 BA, Bilthoven, The Netherlands

**References**

1. Armstrong R, Waters E, Dobbins M, Anderson L, Moore L, Petticrew M, et al. Knowledge translation strategies to improve the use of evidence in public health decision making in local government: intervention design and implementation plan. Implement Sci. 2013;8:121.

2. Faggiano F, Allara E, Giannotta F, Molinar R, Sumnall H, Wiers R, et al. Europe needs a central, transparent, and evidence-based approval process for behavioural prevention interventions. PLoS Med. 2014;11(10):e1001740-e.

3. Ng E, de Colombani P. Framework for Selecting Best Practices in Public Health: A Systematic Literature Review. J Public Health Res. 2015;4(3):577-.

4. Campbell DM, Redman S, Jorm L, Cooke M, Zwi AB, Rychetnik L. Increasing the use of evidence in health policy: practice and views of policy makers and researchers. Aust New Zealand Health Policy. 2009;6:21.

5. Oliver K, Innvar S, Lorenc T, Woodman J, Thomas J. A systematic review of barriers to and facilitators of the use of evidence by policymakers. BMC Health Services Research. 2014;14(1):2.

6. Quinn E, Huckel-Schneider C, Campbell D, Seale H, Milat AJ. How can knowledge exchange portals assist in knowledge management for evidence-informed decision making in public health? BMC public health. 2014;14:443.

7. Barnfield A, Savolainen N, Lounamaa A. Health Promotion Interventions: Lessons from the Transfer of Good Practices in CHRODIS-PLUS. Int J Environ Res Public Health. 2020;17(4).

8. Burkhardt JT, Schröter DC, Magura S, Means SN, Coryn CL. An overview of evidence-based program registers (EBPRs) for behavioral health. Eval Program Plann. 2015;48:92-9.

9. Rhodes T, Lancaster K. Evidence-making interventions in health: A conceptual framing. Social Science & Medicine. 2019;239(112488).

10. EuroHealthNet Webpage [Internet].place unknown: publisher unknown]; [cited 2022 Sept 4] Available from: <https://eurohealthnet.eu/>.

11. Fazal N, Jackson SF, Wong K, Yessis J, Jetha N. Between worst and best: developing criteria to identify promising practices in health promotion and disease prevention for the Canadian Best Practices Portal. Health Promot Chronic Dis Prev Can. 2017;37(11):386-92.

12. Means SN, Magura S, Burkhardt JT, Schröter DC, Coryn CLS. Comparing rating paradigms for evidence-based program registers in behavioral health: evidentiary criteria and implications for assessing programs. Eval Program Plann. 2015;48:100-16.

13. Zack MK, Karre JK, Olson J, Perkins DF. Similarities and differences in program registers: A case study. Eval Program Plann. 2019;76:101676.

14. Brownson RC, Fielding JE, Maylahn CM. Evidence-Based Public Health: A Fundamental Concept for Public Health Practice. Annual Review of Public Health. 2009;30(1):175-201.

15. Buckley PR, Fagan AA, Pampel FC, Hill KG. Making Evidence-Based Interventions Relevant for Users: A Comparison of Requirements for Dissemination Readiness Across Program Registries. Evaluation Review. 2020;44(1):51-83.

16. Rabin BA, Brownson RC, Haire-Joshu D, Kreuter MW, Weaver NL. A glossary for dissemination and implementation research in health. J Public Health Manag Pract. 2008;14(2):117-23.

17Stepien M, Keller I, Takki M, Caldeira S. European public health best practice portal - process and criteria for best practice assessment. Archives of Public Health. 2022;80(1):131.

18 Brug J, van Dale D, Lanting L, Kremers S, Veenhof C, Leurs M, et al. Towards evidence-based, quality-controlled health promotion: the Dutch recognition system for health promotion interventions. Health education research. 2010;25(6):1100-6.

19. Riley T, Jose K, Garvey K, Morgan M. A Systems Approach to the Coproduction of Evidence for Health Promotion. In: Potvin L, Jourdan D, editors. Global Handbook of Health Promotion Research, Vol 1: Mapping Health Promotion Research. Cham: Springer International Publishing; 2022. p. 641-57.

20. Horizon 2021-2027 webpage, European Commsion, cited [ 13-4-2-2023]. Available from [Funding programmes and open calls (europa.eu)](https://research-and-innovation.ec.europa.eu/funding/funding-opportunities/funding-programmes-and-open-calls_en#health)
